# The complete chloroplast genome sequence with a novel 24-bp deletion of a Korean solid green-type cucumber variety (*Cucumis sativus* var. *sativus*)

**DOI:** 10.1080/23802359.2017.1398604

**Published:** 2017-11-06

**Authors:** Sang-Choon Lee, Hyun Oh Lee, Ho Jun Joh, Inseo Kim, Won-Kyung Lee, Tae-Jin Yang, Kihwan Song

**Affiliations:** a Department of Plant Science, Plant Genomics and Breeding Institute, and Research Institute of Agriculture and Life Sciences, College of Agriculture and Life Sciences, Seoul National University, Seoul, Republic of Korea;; b Phyzen Genomics Institute, Seongnam, Gyeonggi-do, Republic of Korea;; c Department of Bioresources Engineering College of Life Sciences, Sejong University, Seoul, Republic of Korea

**Keywords:** *Cucumis sativus*, cucumber, chloroplast genome, novel deletion

## Abstract

Cucumber (*Cucumis sativus* var. *sativus*) is one of the economically important vegetable crops. In this study, we characterized the complete chloroplast genome sequence of inbred line ID YHB bred from Korean solid green-type cucumber variety, through *de novo* assembly using next-generation sequencing. The chloroplast genome is 155,501 bp long and has typical quadripartite structures and gene contents as found in reported cucumber chloroplast genomes. Interestingly, sequence comparison revealed a novel 24-bp deletion present only in the chloroplast genome of the inbred line. Phylogenetic analysis confirmed that the inbred line was closely grouped with cucumber cultivars.

The *Cucumis* genus belongs to the Cucurbitaceae family and consists of about 30 species distributed in tropical and temperate regions (Lu and Jeffrey 2011). Among those species, cucumber is one of the most economically important vegetable crops in the world (Pitrat et al. 1999; Acquaah 2012). Several complete chloroplast genome sequences have been reported in cucumber cultivars (Kim et al. 2006; Plader et al. 2007; Chung et al. 2007). Although various genomic resources have been available for cucumber, the plant species is known to have a narrow genetic base, which makes breeding of this species difficult (Acquaah 2012). On this account, we characterized the complete chloroplast genome sequence of inbred line ID YHB bred from Korean solid green-type cucumber variety.

Genomic DNA was extracted from fresh leaves collected from the inbred line cultivated in a greenhouse of Seoul National University (Seoul, Korea, deposited specimen no. CT604). Illumina paired-end (PE) library with 750-bp insert size was constructed and sequenced using an Illumina MiSeq platform by LabGenomics (www.labgenomics.co.kr, Korea). Complete chloroplast genome sequence was generated by *de novo* assembly using high quality PE reads of about 500 Mb, as described previously (Kim et al. 2015). The chloroplast genome was initially annotated using GeSeq (https://chlorobox.mpimp-golm.mpg.de/geseq-app.html) and manually confirmed using Artemis annotation tool (Rutherford et al. 2000) with BLASTN searches.

Complete chloroplast genome sequence (GenBank accession no. MF095790) of inbred line ID YHB is 155,501 bp long and has typical quadripartite structure consisting of large single copy (LSC) region of 86,877 bp, small single-copy (SSC) region of 18,250 bp, and a pair of inverted repeats (IRa and IRb) of 25,187 bp. The genome contains a total of 115 genes, including 80 protein-coding genes, 31 tRNA genes, and four rRNA genes.

Phylogenetic analysis was carried out based on multiple alignment of complete chloroplast genome sequences and confirmed that inbred line ID YHB was grouped with *Cucumis* species such as cucumber cultivars, wild cucumber and melon, where the line was much closed to another variety of Korean solid green-type cucumber (Figure 1). Interestingly, sequence comparison revealed a novel 24-bp deletion within *ycf1* genes in the YHB chloroplast genome but not in reported chloroplast genomes of other cucumbers. The 24-bp deletion resulted from copy number variation of 24-bp tandem repeats, which was confirmed by PCR amplification and nucleotide sequencing.

**Figure 1.**
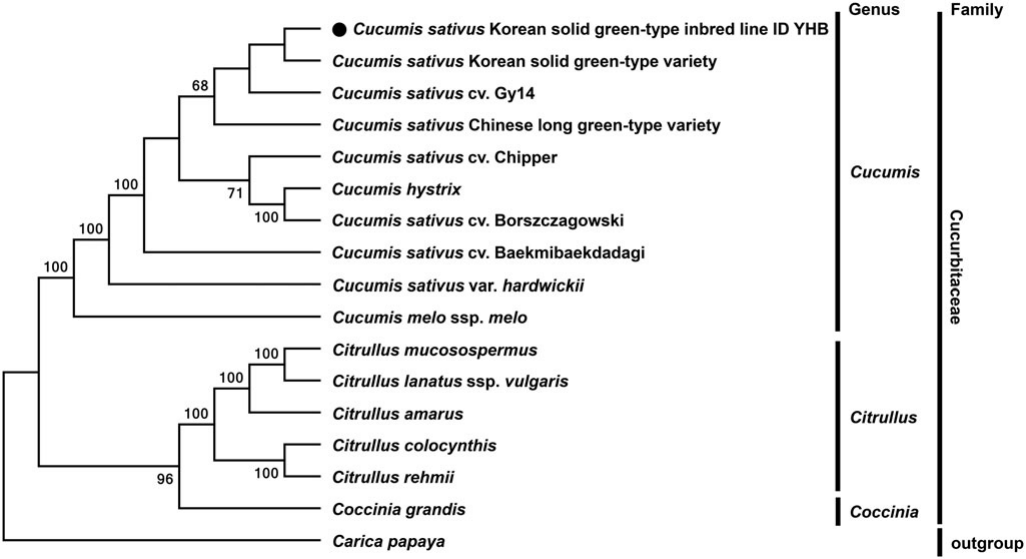
Phylogenetic tree of inbred line ID YHB and other species in the Cucurbitaceae family. Tree was generated by multiple alignment of complete chloroplast genome sequences using MAFFT (http://mafft.cbrc.jp/alignment/server/index.html) and a neighbour-joining (NJ) analysis using MEGA 6.0 (Tamura et al. 2013). Numbers in the nodes are bootstrap support values (>50%) from 1000 replicates. Chloroplast genome sequences used for this tree are: *Cucumis sativus* Korean solid green-type inbred line ID YHB, MF095790; *Cucumis sativus* Korean solid green-type variety, KX231327; *Cucumis sativus* cv. Gy14, DQ865975; *Cucumis sativus* Chinese long green-type variety, KX231328; *Cucumis sativus* cv. Chipper, DQ865976; *Cucumis hystrix*, KF957866; *Cucumis sativus* cv. Borszczagowski, AJ970307; *Cucumis sativus* cv. Baekmibaekdadagi, DQ119058; *Cucumis sativus* var. *hardwickii*, MF536709; *Cucumis melo* ssp. *melo*, JF412791; *Citrullus mucosospermus*, KY430686; *Citrullus lanatus* ssp. *vulgaris*, KY014105; *Citrullus amarus*, MF536694; *Citrullus colocynthis*, MF357889; *Citrullus rehmii*, MF536695; *Coccinia grandis*, KX147312; *Carica papaya*, EU431223.

In conclusion, the chloroplast sequence and polymorphic site identified in this study will contribute to enriching genetic resources of cucumber.
